# Suppression of viral rebound by a Rev-dependent lentiviral particle in SIV-infected rhesus macaques

**DOI:** 10.1038/s41434-024-00467-9

**Published:** 2024-07-18

**Authors:** Brian Hetrick, Summer Siddiqui, Mark Spear, Jia Guo, Huizhi Liang, Yajing Fu, Zhijun Yang, Lara Doyle-Meyers, Bapi Pahar, Ronald S. Veazey, Jason Dufour, Ali Andalibi, Binhua Ling, Yuntao Wu

**Affiliations:** 1 Center for Infectious Disease Research, George Mason University, Manassas, VA 20110 USA; 2 Tulane National Primate Research Center, Tulane University School of Medicine, Covington, LA 70433 USA; 3 Present Address: Integrated Research Facility at Fort Detrick, National Institute of Allergy and Infectious Diseases, National Institutes of Health, Fort Detrick, Frederick, MD USA; 4 Present Address: Host-Pathogen Interaction Program, Texas Biomedical Research Institute, 8715 W Military Dr., San Antonio, TX 78227 USA

**Keywords:** Virology, Infectious diseases, Genetic vectors

## Abstract

Persistence of human immunodeficiency virus (HIV) reservoirs prevents viral eradication, and consequently HIV-infected patients require lifetime treatment with antiretroviral therapy (ART) [1–5]. Currently, there are no effective therapeutics to prevent HIV rebound upon ART cessation. Here we describe an HIV/SIV Rev-dependent lentiviral particle that can be administered to inhibit viral rebound [6–9]. Using simian immunodeficiency virus (SIV)-infected rhesus macaques as a model, we demonstrate that the administration of pre-assembled SIV Rev-dependent lentiviral particles into SIVmac239-infected Indian rhesus macaques can lead to reduction of viral rebound upon ART termination. One of the injected animals, KC50, controlled plasma and CNS viremia to an undetectable level most of the time for over two years after ART termination. Surprisingly, detailed molecular and immunological characterization revealed that viremia control was concomitant with the induction of neutralizing antibodies (nAbs) following the administration of the Rev-dependent vectors. This study emphasizes the importance of neutralizing antibodies (nAbs) for viremia control [10–15], and also provides proof of concept that the Rev-dependent vector can be used to target viral reservoirs, including the CNS reservoirs, in vivo. However, future large-scale in vivo studies are needed to understand the potential mechanisms of viremia control induced by the Rev-dependent vector.

HIV infection is currently treatable but not curable [1, 16–18]. Anti-retroviral therapy (ART) can effectively suppress viral replication, but requires lifelong treatment that is associated with adverse drug effects [19] and viral persistence [3, 4]. A major hurdle preventing an effective or functional cure is the existence of viral reservoirs [5, 20, 21], which produce viral rebound upon ART cessation. Various experimental approaches have been in development to diminish viral rebound by either reactivating (“shock and kill”) [22] or suppressing (“block and lock”) viral reservoirs [23, 24]. A major challenge has been the inability to selectively target viral reservoirs in vivo [22, 25]. Alternative approaches have also been tested for long-term control of viral rebound. For example, as a proof of concept, it was recently shown that the delivery of anti-HIV monoclonal antibodies through an adeno-associated virus vector can drive long-term virologic suppression in an SHIV-infected monkey [26].

For targeting HIV-infected cells, we previously developed an HIV Rev-dependent lentiviral vector that can selectively express genes only in HIV+ cells [6–8, 27]. HIV Rev is a virus-encoded early protein that regulates the splicing and nuclear export of unspliced and singly-spliced viral mRNAs [9, 28]. Rev binds directly to a regulatory element, the Rev-responsive element (RRE), on the unspliced and singly-spliced viral mRNAs [29], and mediates viral mRNA nuclear export [9], polysomal association for protein translation [30–32], and viral genomic RNA dimerization for packaging [33–35]. The essential role of Rev has led us to use the Rev-RRE interaction as a regulator to achieve selective expression of reporter or therapeutic genes only in HIV-infected cells where Rev is expressed [6, 27]. The high selectivity and stringency of the Rev-dependent gene expression has been demonstrated in studies showing that reporter expression from the Rev-dependent vector is strictly dependent on the presence of HIV, and is not responsive to cellular stimuli such as cytokines and mitogens [6, 27, 36–38]. The Rev-dependent vector has also been tested in vitro in HIV+ cells for selective expression of therapeutic genes such as *anthrolysin* O (AlnO) from *Bacillus anthracis*, diphtheria toxin A chain (DT-A) from *Corynebacterium diphtheriae*, the human pro-apoptotic gene TRAF6 (tumor necrosis factor receptor-associated factor 6), and the thymidine kinase gene from herpes simplex 1 virus (HSV1-tk) [7, 8]; these therapeutic genes, which induce cell death by different mechanisms, have been shown to selectively kill HIV-infected T cells and macrophages when delivered via the Rev-dependent vector [7, 8]. Here we report the first in vivo study using the Rev-dependent vector to target viral reservoirs in SIVmac239-infected Indian rhesus macaques.

We constructed an SIV Rev-dependent vector based on our previously reported HIV Rev-dependent vector [6] (Fig. 1a). SIV encodes the Rev protein and possesses Rev functionality, which is well-conserved among HIV and SIV strains. In fact, it has been shown that SIV Rev is capable of inducing cytoplasmic expression of incompletely spliced mRNAs [39], and that the Rev proteins of HIV-1 and HIV-2 can also transactivate SIV RRE-dependent gene expression [40]. For the SIV Rev-dependent vector, we converted all HIV-based elements into homologous SIV sequences from the SIVmac239 genome, including the multiple splicing sites (D1, A6, D3, A7), RRE, and Loop A (ψ). The vector was validated by cloning two reporter genes, GFP and LacZ, into the SIV Rev-dependent vector, and we observed the expression of the reporter genes following co-transfection, particle assembly, and infection of SIV+ cells (Fig. 1b, e, f). For SIV Rev-dependent expression of therapeutic genes, we also cloned the thymidine kinase gene from herpes simplex 1 virus (HSV-1-tk) and the human TRAF6 gene into the SIV-based vector (Fig. 1b). To assemble viral particles, we used the SIV-based helper vector, pAD-SIV3+ [41, 42], pSIV-Nef [43], and an SIV gp160-expressing vector (pLTR-SIV-Env). These vectors were co-transfected into HEK293T cells to assemble into viral particles that were subsequently purified and concentrated 500- to 1000-fold through anion exchange and size-exclusion columns, and then used for injection into animals (Fig. 1c, d). For the initial safety assessment, all concentrated SIV Rev-dependent particles were injected into mice, and we did not observe adverse effects (Supplementary Fig. S1). The concentrated SIV Rev-dependent TRAF6(R) particles were also injected into two rhesus macaques. Both animals appeared healthy with no noticeable adverse effects and were euthanized after 6 months to collect tissues for toxicity examination. Analysis of multiple tissues did not show any differences between the injected animals and a normal control animal, as shown in the representative histological specimens from the kidney and the liver (Fig. 1g).

**Fig. 1:**
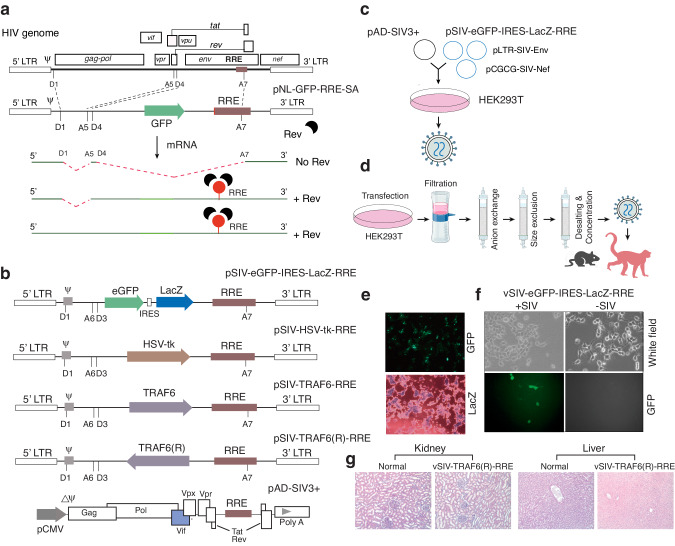
Development of the SIV Rev-dependent vectors to target SIV reservoirs. **a** Illustration of the HIV Rev-dependent vector, pNL-GFP-RRE-SA. Shown are the packaging signal (ψ), RRE, and the splicing donors (D1, D4) and acceptors (A5, A7) and their involvement in splicing. In the absence of HIV Rev, all transcripts are spliced and the GFP ORF is removed, no GFP expression. In the presence of Rev, Rev binds to RRE, preventing splicing, and mediating the nuclear export and translation of the unspliced and singly-spliced GFP-containing mRNAs. GFP is expressed. **b** The SIV Rev-dependent vectors, which are constructed based on the HIV Rev-dependent vector, carry reporter or therapeutic genes (GFP, lacZ, HSV-1-tk, or TRAF6). The helper plasmid pAD-SIV3+ was used for particle assembly. **c**, **d** To assemble virus particles, individual SIV Rev-dependent vector was co-transfected with pAD-SIV3+ plus pLTR-SIV-Env and pCGCG-SIV-Nef into HEK293T cells, and viruses were harvested at 48 h post co-transfection, and then purified and concentrated. **e**, **f** Expression of reporter genes in HEK293T cells co-transfected with pSIV-eGFP-IRES-LacZ-RRE (**e**). vSIV-eGFP-IRES-LacZ-RRE viral particles were assembled and used to infect SIV+ cells. GFP expression was observed (**f**). **g** Particles were intravenously injected into two rhesus macaques. At 6 months post injection, tissue biopsy was conducted on one of the monkeys (BR85). Shown are the comparisons of kidney and live histology between the normal and the injected animal. The kidney renal architecture and cell morphology appear normal. The hepatic architecture and cell morphology also appear normal.

To test possible in vivo therapeutic effects of the Rev-dependent vector for targeting SIV infection, we chose HSV-1-tk as the therapeutic gene. Our selection of HSV-1-tk was based on two factors. Firstly, HSV-1-tk/nucleoside analog-mediated cell killing has been studied for over 30 years [44–48] and has been tested in several clinical trials, demonstrating desirable safety and efficacy [48–50]. Secondly, the lack of cytotoxicity of HSV-1-tk in the absence of ganciclovir (GCV) permits the production of high-titer lentiviral particles (e.g., >10^10^ transduction units) for animal injection [49, 50]. In contrast, the low-level cytotoxicity of TRAF6 in HEK293T cells makes it difficult to produce high-titer vSIV-TRAF6-RRE particles, which requires a greater degree of particle concentration.

To conduct the animal trial, Indian macaques were divided into 4 groups (Groups A to D, two animals per group) (Fig. 2a). For Group A, monkeys were infected with 100 TCID_50_ of SIVmac239 using our standard infection protocol [51–53] but received no ART. For Group B, animals were similarly infected with SIV but were treated with ART for 11 months. ART was then terminated to allow viral rebound to occur. For Group C, animals were infected and treated with ART for 11 months. ART was then stopped, and animals were injected with 0.5–1 ml of concentrated vSIV-HSV-1-tk-RRE particles. ART was not resumed in the animals after vSIV-HSV-1-tk-RRE injection. This was to permit vector amplification and mobilization with active SIV replication. Group D animals were treated and injected similarly to Group C, but ART was resumed for 3 months after vector injection. The scheduled ART interruption during lentiviral vector injection is needed for vSIV-HSV-1-tk-RRE infection, vector amplification, and mobilization in the body, which normally peak in 7–10 days [54]. The Rev-dependent vector contains fully functional LTR, the packaging signal (Ψ), and RRE, which can co-replicate and spread from cell to cell with SIV, similar to the mobilization of a lentiviral vector with HIV [54]. The resumption of ART after vector injection in Group D was to inhibit uncontrolled SIV spread. ART was terminated after 3 months, and GCV was administered intravenously for two weeks at a dosage of 10 mg/kg/day to induce the killing of SIV+ cells by HSV-1-tk for animals in Groups C and D. Following GCV administration, all treatments were terminated and viral rebound was monitored.

**Fig. 2:**
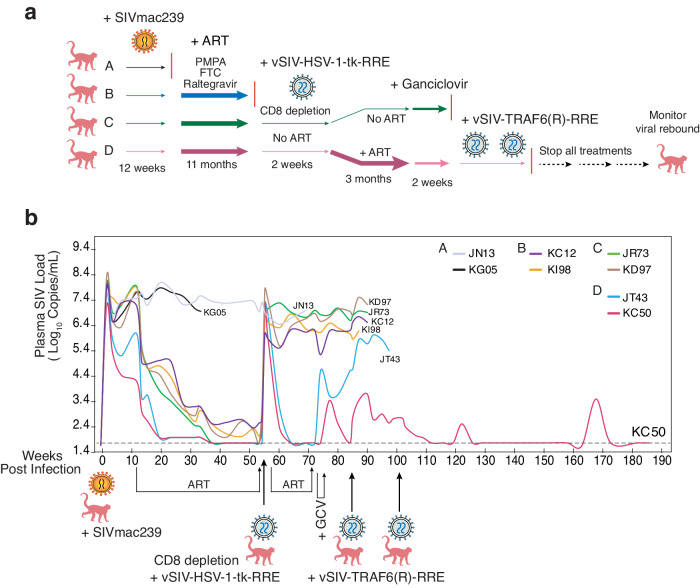
In vivo testing of the SIV Rev-dependent vectors in SIVmac239-infected Indian rhesus macaques. **a** Schematic of the different treatments and vector injection designs for study Group A–D (*n* = 2 for each group). **b** Plasma viral loads (copies/ml) following SIVmac239 infection, ART, vSIV-HSV-1-tk-RRE/GCV injection, and vSIV-TRAF6(R)-RRE injection in animals of Group A–D.

For the animals in Groups C and D, before the vector injection, CD8 T cells were also transiently depleted with the administration of an anti-CD8 antibody, cM-T807 [55]. A major rationale for this transient CD8 T cell depletion is to prevent possible rapid clearance of cells carrying the Rev-dependent vector. CD8 T cells are normally regenerated after a few months [55]

The experiment results are summarized in Fig. 2b. As expected, animals in Group A had high viral loads without ART. In Group B, ART diminished viral loads that rebounded quickly following ART termination. For animals in Group C, the injection of vSIV-HSV-1-tk-RRE/GCV failed to inhibit viral rebound following ART termination, suggesting that the amount of vector injected was not capable of competing with uncontrolled SIV replication. However, for the animals in Group D, the administration of vSIV-HSV-1-tk-RRE/GCV inhibited viral rebound following ART termination (Fig. 2b). One of the two animals, KC50, had viral loads diminished to the baseline level with vSIV-HSV-1-tk-RRE/GCV treatment.

We repeated the Group D therapeutic regimen in four additional animals. As shown in Supplementary Fig. S2, HSV-1-tk/GCV-injection kept viral rebound to below 10^4^-10^5^ with ART termination. In addition, after stopping GCV and all other treatments, viral loads were self-controlled 3-4 logs below the peak viral loads (10^7^-10^8^) over the course of 10 weeks, demonstrating the ability of the Rev-dependent vector to reduce viral rebound. However, the inability of vSIV-HSV-1-tk-RRE/GCV to reduce viral loads to baseline levels was likely an outcome of partial targeting of reservoir cells. We attempted to reduce viral loads further by using an additional vector, vSIV-TRAF6-RRE, and subsequently injected the animals with the particle. We hoped that this vector would be stronger, which might aggressively deplete SIV+ cells with the expression of cytotoxic TRAF6. Unexpectedly, two of the animals (LB29 and KC81) showed severe adverse effects and succumbed within hours of the vSIV-TRAF6-RRE particle injection.

Given that it would be difficult to superinfect and kill all reservoir cells, we were interested in determining whether we could use the Rev-dependent vector to partially target reservoir cells by converting at least some of them to release non-infectious particles to stimulate immunity. This should be achievable. To this end, we chose to test an additional vector and subsequently injected animals with a second Rev-dependent particle, vSIV-TRAF6(R)-RRE, which does not express a functional gene with the TRAF6 in reverse direction (Fig. 1). We also decided to follow the more responsive animal, KC50, in Group D for a long-term study. The vSIV-TRAF6(R)-RRE vector can reduce the release of infectious particles through competing for Tat, Rev, and genomic packaging in reservoir cells. As shown in Fig. 2b, vSIV-TRAF6(R)-RRE administration diminished viral rebound, likely through vector mobilization and competition with residual viruses and/or stimulation of immune control, as a result of releasing non-infectious, immunogenic particles from reservoir cells. Following the first vSIV-TRAF6(R)-RRE injection, we gave the animal a second batch injection of vSIV-TRAF6(R)-RRE four months later. The animal received no further treatment following this injection, which resulted in a further reduction of viral loads to undetectable levels. Surprisingly, KC50 was able to self-control viral rebound, and stably maintained viral load to undetectable levels most of the time with only two self-contained viral load blips.

To confirm the results obtained from quantifying peripheral blood viremia, we also quantified viral loads in the cerebrospinal fluid (CSF) (Fig. 3). CSF samples for Group D were collected approximately every 20 weeks. KC50 and JT43 both had detectable levels of SIV in their CSF before any SIV Rev-dependent particle injection. JT43 showed decreased CSF viral load after administration of each of the SIV Rev-dependent vector treatments. KC50 maintained no detectable CSF viral load after the first vector injection, demonstrating that viremia control extends to the CSF.

**Fig. 3:**
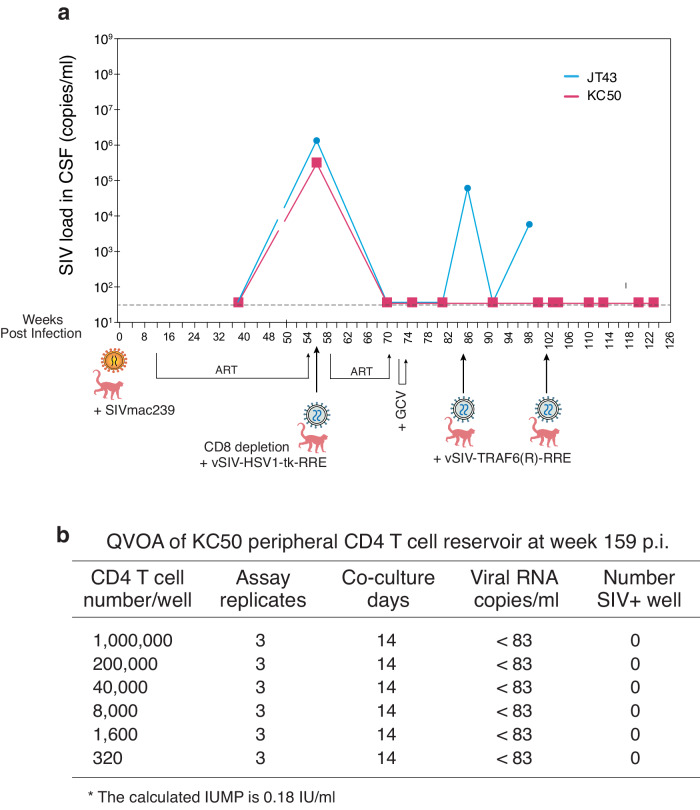
Quantification of CSF viral loads and peripheral CD4 T cell reservoirs. **a** Quantification of CSF viral loads in animals treated with the Rev-dependent vector (Group D). **b** Quantitative viral outgrowth assay (QVOA) of peripheral blood CD4 T cells from KC50. PBMC were collected at week 159 from KC50, and CD4 T cells were purified, activated with PHA plus IL-2. Cells were serially diluted (5 fold dilution), and mixed with CEMx174 cells (1 × 10^5^) and co-cultured for two weeks. The culture supernatant were collected and quantified by SIV RNA using qRT-PCR. The qRT-PCR limit of detection is 83 copies/ml. IUMP (Infectious Units Per Million) was calculated.

We further quantified the CD4 T cell reservoirs in the peripheral blood of KC50 using a quantitative viral outgrowth assay (QVOA). Peripheral blood mononuclear cells (PBMC) were collected from KC50 at week 159, and CD4 T cells were purified, serially diluted (5-fold, starting at 1 million cells), activated, and then co-cultured with CEMx174 cells for two weeks for virus expansion. Culture supernatants were quantified for cell-associated (CA) SIV RNA using qRT-PCR. As shown in Fig. 3b, we did not detect CA SIV RNA in all dilutions, demonstrating the diminishment of viral reservoirs in the peripheral blood CD4 T cells. However, it is likely that tissue reservoirs still existed, as KC50 had two viral load blips in plasma (Fig. 2b).

The long-term viremia remission in KC50, both in the peripheral blood and in CSF, is surprising and encouraging since the animal was infected with the most pathogenic strain of SIV, SIVmac239, and is also negative for Mamu-A*01, B*08 and B*17 expression. To determine whether the injection of the SIV Rev-dependent vectors can induce the production of neutralizing antibodies (nAb), a pseudovirus-based antibody neutralization assay was performed using the envelope from the neutralization-resistant, tier 2 strain SIVmac239CS.23. A pseudovirus bearing the neutralization-sensitive tier 1 strain SIVmac251.6 envelope was also used for comparison. In addition, an MLV-pseudotyped virus was used as a negative control. No neutralization was detected against the MLV-pseudotyped virus in KC50, while high levels of neutralization of the tier 1 pseudovirus SIVmac251.6 were detected after the vSIV-HSV-1-tk-RRE injection (Fig. 4a). We also observed neutralization of the tier 2 pseudovirus SIVmac239CS.23, which began at weeks 55–60 after vSIV-HSV-1-tk-RRE injection (Fig. 4b). The level of neutralizing antibodies gradually increased following vSIV-TRAF6(R)-RRE injection. The titers of the neutralizing antibodies continued to increase up to 4-fold over the course of 48 weeks after the second injection of vSIV-TRAF6(R)-RRE. The presence of high titers of neutralizing antibodies was coincident with the period when plasma viremia remained undetectable. These results suggest that targeting viral reservoir cells with the Rev-dependent vector may induce highly active neutralizing antibodies, which could, in turn, help to control plasma viremia.

**Fig. 4:**
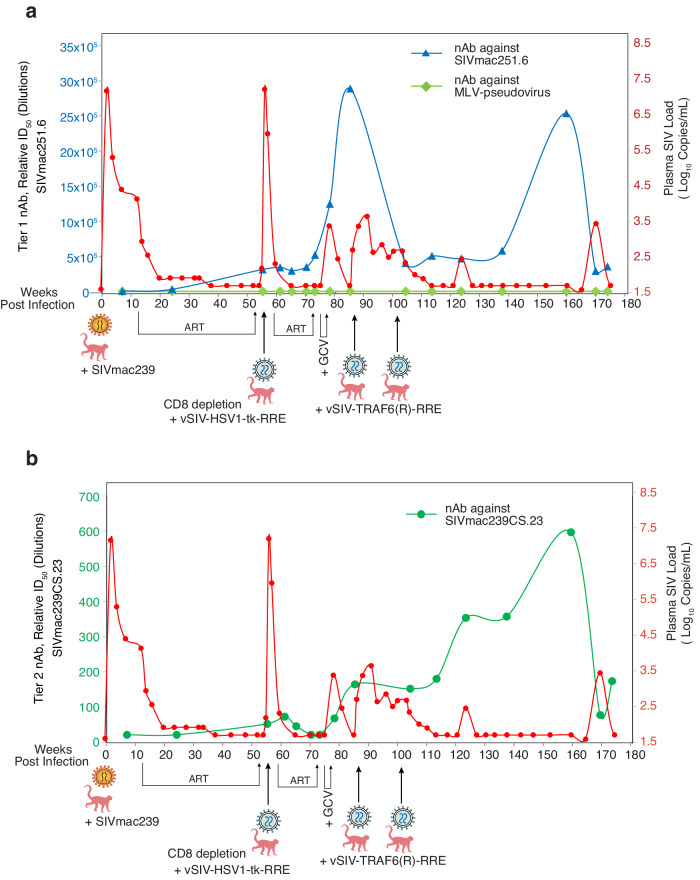
Quantification of neutralizing antibodies in KC50. Quantification of neutralizing antibodies was conducted using a pseudovirus-based antibody neutralization assay, which utilizes a pseudovirus carrying the envelope of the tier 1 strain SIVmac251.6 or an MLV-pseudotyped virus as a negative control (**a**). A pseudovirus carrying the envelope of the tier 2 strain SIVmac239CS.23 was also quantified (**b**). The ID_50_ values represent serum dilutions at which 50% neutralization occurred. For comparison, plasma viral loads in KC50 were also plotted (red line). The background signals against MLV-pseudovirus are all <20. Positive for neutralizing antibody activity is based on the criterion of ≥3× signal of the MLV control.

We have previously demonstrated the capacity of the HIV Rev-dependent vector for selective targeting and killing of HIV + CD4 T cells and macrophages in vitro [6–8]. Here, we report our first proof-of-concept animal studies of the Rev-dependent vector for targeting viral reservoirs, in which we observed the capacity of the vector to reduce viral rebound in vivo. One of the treated animals, KC50, controlled plasma viremia to an undetectable level most of the time for two years after ART termination. The remission of plasma and CNS viremia to an undetectable level in KC50 was unexpected. KC50 was infected with the most pathogenic SIV, SIVmac239, and is also negative for Mamu-A*01, B*08 and B*17 expression [56]. In addition, KC50 did not show spontaneous viremia control after the termination of ART, and viral rebound reached high levels prior to the injection of the Rev-dependent particles (Fig. 2). Control of viremia to undetectable level was achieved only after the multiple injections of the Rev-dependent particles. Given the difficulty of identifying and targeting reservoir cells in the body, we expected that the Rev-dependent vector would only be able to target reservoirs partially, even after in vivo vector amplification and mobilization. Complete elimination of viral reservoirs is unlikely, as it would require superinfection of essentially every HIV/SIV+ cell in the body. As such, the mechanisms of viremia control to the limit of detection in KC50 are not fully understood, and likely a combined result of multiple processes. The initial amplification and mobilization of the SIV-HSV-1-tk-RRE vector followed by GCV-mediated cell killing may reduce viral reservoirs sufficiently to allow the induction of highly effective tier 2 neutralizing antibodies for viremia control. The nAbs produced initially from SIV infection are not capable of controlling viremia to a low level. Reducing reservoir cells with the Rev-dependent vector followed by subsequent restimulation may allow the induction of a second wave of nAbs capable of greater viremia control. It has been shown that the increase in the neutralization breadth of nAbs correlates with the duration of infection, but is independent of properties of viral inoculum, viral loads, or viral diversity [57]. It is possible that persistent production of non-infectious particles from reservoir cells following the Rev-dependent vector injections may increase the breadth of neutralizing antibodies. Importantly, nAbs have been shown to be capable of greatly reducing plasma viremia and viral reservoirs when passively administered [10–15]. Nevertheless, for most HIV-infected patients, nAbs naturally induced are only capable of neutralizing their homologous viruses, and only a few elite neutralizers can develop broad nAbs [58]. The Rev-dependent vector may have the potential to induce nAbs with expanded breadth in patients.

The infection and the mobilization of the Rev-dependent vector among memory T cells and macrophages may allow the vector to be carried into sanctuary sites such as lymphoid tissues and CNS that are normally difficult to access for therapeutics. GCV has been shown to cross the blood-brain barrier (BBB) [59], which may allow CNS-specific targeting of SIV-infected cells. Notably, targeted killing of tumor cells in the brain has been observed after an HSV-tk vector intrathecal injection and systemic GCV administration in rats [60].

Our study did not quantify anti-SIV CD8 T cells stimulated by the Rev-dependent particles. We expect, however, that CD8 T cells likely play an important role in suppressing viral reservoirs and rebound [61]. In our study, CD8 T cells were transiently depleted during particle injection, likely resulting in reservoir reactivation [61]. This may have allowed a greater degree of vector amplification and mobilization among reservoir cells.

Blood resting memory CD4 T cells are a major HIV reservoir [20]. In addition, terminally-differentiated HIV+ macrophages resist virus-mediated apoptosis and serve as persistent viral producer cells [62]. HIV+ blood CD4 T cells and macrophages have been shown to be targeted and selectively killed by the Rev-dependent vector in vitro [7, 8]. GCV has also been shown to be able to target post-mitotic cells and induce the killing of terminally-differentiated synovial lining cells following HSV-tk injection in a rabbit inflammatory arthritis model [63]. It is possible that the inhibition of viral rebound in the CNS in our study is mediated by the Rev-dependent vector carried into the brain by macrophages.

In our study, the dramatic decrease of viremia in KC50 occurred after the injection of the Rev-dependent particles and the induction of high-titer tier 2 nAb. As shown in Fig. 4a, the tier 1 nAB increased following the first particle injection, whereas the 2nd and 3rd injection did not immediately induce tier 1 nAB. This delayed, second peak of tier 1 nAB overlaps the tier 2 nAB observed in Fig. 4b. It is likely that this second peak of nAB (Fig. 4a) reflects the induction of the tier 2 nAB. Previous studies have shown that the induction of broad nAbs correlates with the duration of infection, but is independent of properties of viral inoculum, viral loads, or viral diversity [57]. Thus, it is likely that the longer time observed for the induction of the second peak in Fig. 4a may reflect the time needed for the induction of the tier 2 nAB. Although the mechanism of nAb induction by the Rev-dependent vectors was not studied in detail, superinfection of reservoir cells by the Rev-dependent particles likely turns them into cells persistently releasing defective viruses; vSIV-TRAF6(R)-RRE does not express a functional gene, but its defective mini-genome is fully capable of competing for Tat and Rev and being co-packaged into SIV for mobilization. Persistent release of such defective particles from reservoir cells can certainly serve to stimulate antiviral immunity and induce the production of neutralizing antibodies [57]. Thus, our study suggests a possible new strategy of “rehab and redeem” that uses the Rev-dependent lentiviral vector to convert reservoir cells to release non-infectious particles that stimulate the production of nABs. A major advantage of this strategy is that it does not require the targeting of all reservoir cells. As long as strong nAbs are induced by partial reservoir targeting, patients may be able to self-control plasma viremia for years, as demonstrated in KC50 in our study.

There are several limitations of our studies. Firstly, as a proof-of-concept study, animals were injected only once with the therapeutic vSIV-HSV-1-tk-RRE particle, and only one animal, KC50, was administered with a regimen of two doses of the vSIV-TRAF6(R)–RRE vector. In future studies, more animals with multiple injections are needed to study the mechanisms of viremia control, and to draw statistically significant conclusions for the efficacy of the Rev-dependent vector in targeting viral reservoirs in vivo. Secondly, we did not quantify cell-mediated immune responses (CMI). Control of viremia as shown in KC50 could have been a combined effect of nAB and CMI, as CD8 T cells have been shown to play an important role in suppressing viral reservoirs and rebound [61]. In future studies, CMI needs to be evaluated. Thirdly, although we quantified tier 1 and tier 2 nABs against SIVmac251.6 and SIVmac239CS.23, respectively, we did not quantify nAB against multiples viral strains to determine the breadth of nABs in KC50. Future studies need to quantify broad nAB (bnAB) using a panel of viral strains. In addition, the severe adverse effects observed from the use of the vSIV-TRAF6-RRE vector were not fully studied. We suspected that it might be related to the presence of high levels of TRAF6 or other human proteins in the particles during assembly, which may have triggered strong immune responses during injection. Because of the cytotoxicity of TRAF6, the particles produced from co-transfection have a much lower titer and need to be highly concentrated [8], which may lead to the enrichment of human proteins.

## Methods

### Ethics statement

Indian-origin rhesus macaques were housed at the Tulane National Primate Research Center (TNPRC), Covington, LA, and BALB/cJ mice were housed at the Biomedical Research Laboratory at George Mason University. Animals were maintained in accordance with Association for Assessment and Accreditation of Laboratory Animal Care International standards. All procedures were reviewed and approved by the Tulane University Institutional Animal Care and Use Committee (IACUC) and by the George Mason University IACUC under protocol number P0254R. All clinical procedures were carried out under the supervision of a laboratory animal care veterinarian. All procedures were carried out under anesthesia using ketamine, and all efforts were made to minimize stress, improve housing conditions, and provide enrichment opportunities.

### Animals and virus infection

Indian-origin rhesus macaques (Macaca mulatta) were utilized in this study. Adult animals with randomly assigned males and females aged in the range of 3–6-year-old were used. The animals were divided into 4 groups: Group A, SIV-infected, not treated (*n* = 2); Group B, SIV-infected and ART-treated (*n* = 2); Group C, SIV-infected, ART-treated, and received vSIV-HSV-1-tk-RRE injection (*n* = 2); Group D, SIV-infected, ART-treated, and received vSIV-HSV-1-tk-RRE injection as in Group C, but ART treatment was resumed after particle injection. Group D animals also received vSIV-TRAF6(R)-RRE injection (*n* = 2). An additional 4 macaques were infected and treated similarly to Group D animals until week 65. Macaques were intravenously inoculated with 100 TCID_50_ SIVmac239 before any treatment (Supplementary Fig. S2).

### Antiretroviral therapy

Animals were treated with ART which includes beta-2′,3′-dideoxy-3′-thia-5-fluorocytidine (FTC, emtricitabine; 40 mg/kg), the nucleoside reverse transcriptase inhibitor (R)-9-(2-phosphonomethoxypropyl)adenine (PMPA, tenofovir; 20 mg/kg; graciously provided by Gilead Sciences) daily by subcutaneous injection, and the integrase inhibitor raltegravir (20 mg/kg; graciously provided by Merck & Co., Inc.) orally twice daily for up to 11 months.

### In vivo depletion of CD8 T cells

Rhesus macaques received a single dose of 50 mg/kg IgG1 recombinant anti-CD8 monoclonal antibody cM-T807, intravenously. cM-T807 was obtained from the NHP Reagent Resource.

### Sample collection

Blood was collected weekly to monthly, and CSF, lymph node, and rectal biopsies were collected before and after infection, during ART treatment, ART termination, CD8 depletion, vSIV-HSV-1-tk-RRE/Ganciclovir injection, and vSIV-TRAF6(R)-RRE injection. Animals were euthanized for tissue collection at the end of the studies.

### Quantification of SIV RNA in plasma and CSF

Plasma samples were obtained from 10 ml EDTA-whole blood by centrifugation at 1500 rpm for 10 min and stored at −80 °C until use. SIV viral RNA was extracted from the plasma and CSF samples using the High Pure Viral RNA Kit (Roche; Indianapolis, IN, USA). The SIV viral loads targeting the conserved gag region of SIVmac239 and SIVmac251 were measured by real-time quantitative PCR (qPCR) assay by the Pathogen Detection and Quantification Core (PDQC) of Tulane National Primate Research Center. Measurements were taken from distinct samples. Plasma and CSF viral loads were determined with a limit of detection of 83 copies/ml.

### Resting CD4 T cell isolation and purification from blood

PBMCs (post infection week 159) were purified from whole blood via Ficoll-paque gradient centrifugation. The CD4 T cells isolated from PBMCs were negatively selected to remove CD8 T cells, B cells, monocytes, NK cells, and granulocytes cells using a non-human primate microbeads CD4 T cell isolation kit (Miltenyi Biotec, Auburn, CA, USA), which includes a cocktail of biotin-conjugated antibodies and anti-biotin magnetic microbeads. The purified CD4 T cells were further separated using anti-CD25 and anti-HLA-DR antibodies for resting CD4 T cells of non-human primate microbeads. The resting CD4 T-cell population generally reached >95% purity.

### Quantitative viral outgrowth assay (QVOA)

Purified resting CD4 T cells were activated with 0.5 μg of PHA/ml to stimulate virus production from latently infected cells. On day 1, the purified CD4 T cells were counted and resuspended to 1 × 10^6^ cells/ml in PHA-containing medium. Purified cells were cultured using 5-fold limiting dilution in duplicate, ranging from 1 × 10^6^ to 3.2 × 10^2^ cells/ml, respectively. On day 2, the medium containing PHA was replaced with fresh medium containing IL-2, and CEMx174 cells (1 × 10^5^) were added and co-cultured for two weeks as previously described [64]. The culture supernatants were collected weekly, and fresh medium was added to the culture. The cell-associated SIV RNA levels in the supernatant were quantified using qRT-PCR. Measurements were taken from distinct samples.

### Cells and vectors

HEK293T cells (ATCC, Manassas, VA, USA) were maintained in DMEM with 10% FBS. The SIV Rev-dependent vectors were synthesized by Genewiz (South Plainfield, NJ, USA). pAD-SIV3+ was kindly provided by Andrea Cara, François-Loïc Cosset, and Didier Negre. pCGCG-Nef plasmid was kindly provided by David Evans. pNL-SIVmac239Env was synthesized by Genewiz.

### Particle assembly

SIV Rev-dependent particles were produced by co-transfection of 5 µg of the SIV Rev-dependent plasmid, 2.5 µg of pAD-SIV3+, 1.25 µg of pCGCG-NEF plasmid, and 1.25 µg of pNL-SIVmac239Env. The transfections were performed using Transfectin (Virongy). Particles were harvested at 48 hours post transfection, filtered through a 0.45 µm filter, purified in anion exchange columns, and concentrated with Vivaspin20 100 K MW columns (Fisher Scientific). The concentrated particles were desalted by washing with 10 volumes of sterile H_2_O.

### SIV neutralizing antibody assay

The neutralizing antibody assays were performed at the Duke Human Vaccine Institute of Duke University. Pseudoviruses enveloped with the neutralization-sensitive Env, SIVmac251.6, and a neutralization-resistant Env, SIVmac239CS.23, were incubated with serial diluted animal serum from multiple weeks post infection. The incubated particles and serum samples were applied to the TZM-bl target cells. As a negative control, these samples were assayed against MLV-pseudotyped virus to give an estimate of non-SIV-specific inhibitory activity in the samples. The relative infectivity was determined by luciferase activity.

## Supplementary information

Supplemental Materials

## Data Availability

Data generated or analyzed during this study can be found within the published article and its supplementary files
