# Supplementary material for: Suppression of viral rebound by a Rev-dependent lentiviral particle in SIV-infected rhesus macaques
Source: Gene Ther. 2024 Jul 18;32(1):16–24. doi: 10.1038/s41434-024-00467-9 (PMC11785524; doi:10.1038/s41434-024-00467-9)
Supplement: Supplementary file 1 — Supplemental Materials [file 41434_2024_467_MOESM1_ESM.docx]

Suppression of viral rebound by a Rev-dependent lentiviral particle in SIV-infected rhesus macaques

Brian Hetrick, Summer Siddiqui, Mark Spear, Jia Guo, Huizhi Liang, Yajing Fu, Zhijun Yang, Lara Doyle-Meyers, Bapi Pahar, Ronald S. Veazey, Jason Dufour, Ali Andalibi, Binhua Ling Yuntao Wu

**SUPPLEMENTARY INFORMATION**


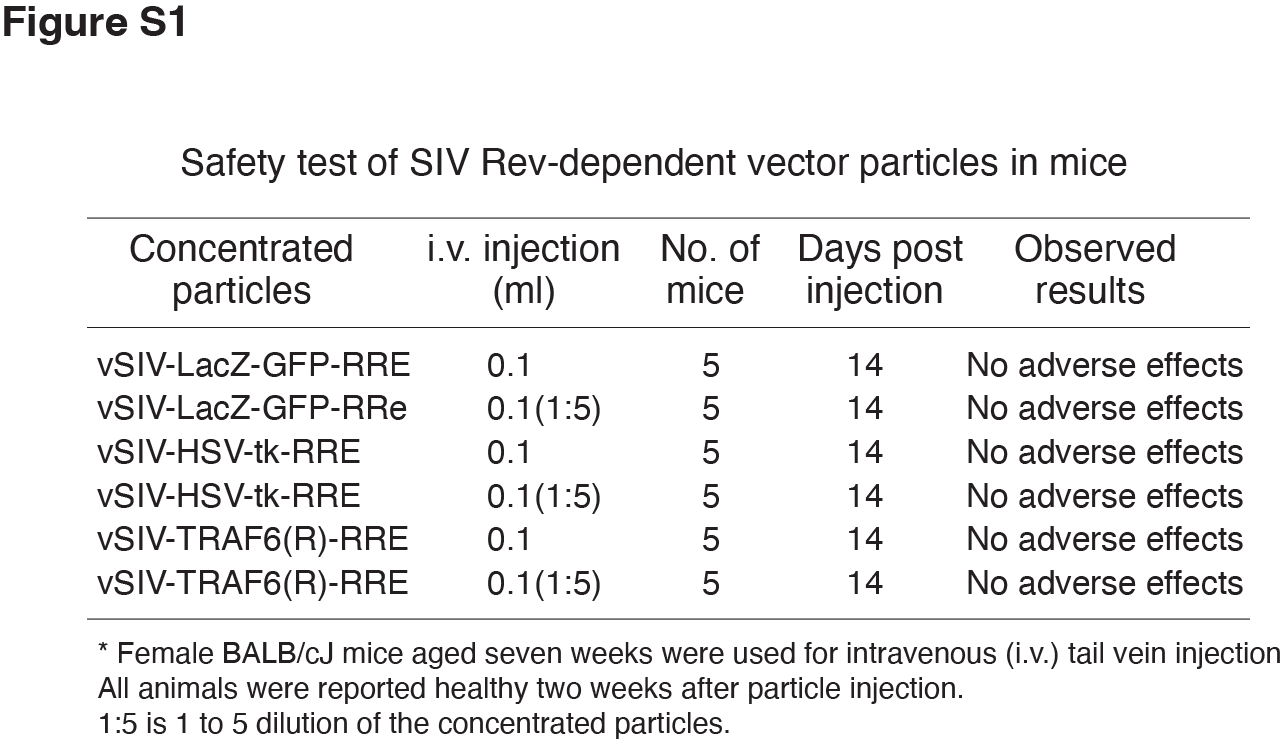


**Figure S1. *In vivo* safety testing of the SIV Rev-dependent vectors in mice.** Female BALB/cJ mice aged seven weeks were used for intravenous (i.v.) tail vein injection. Animals were followed for two weeks after particle injection.


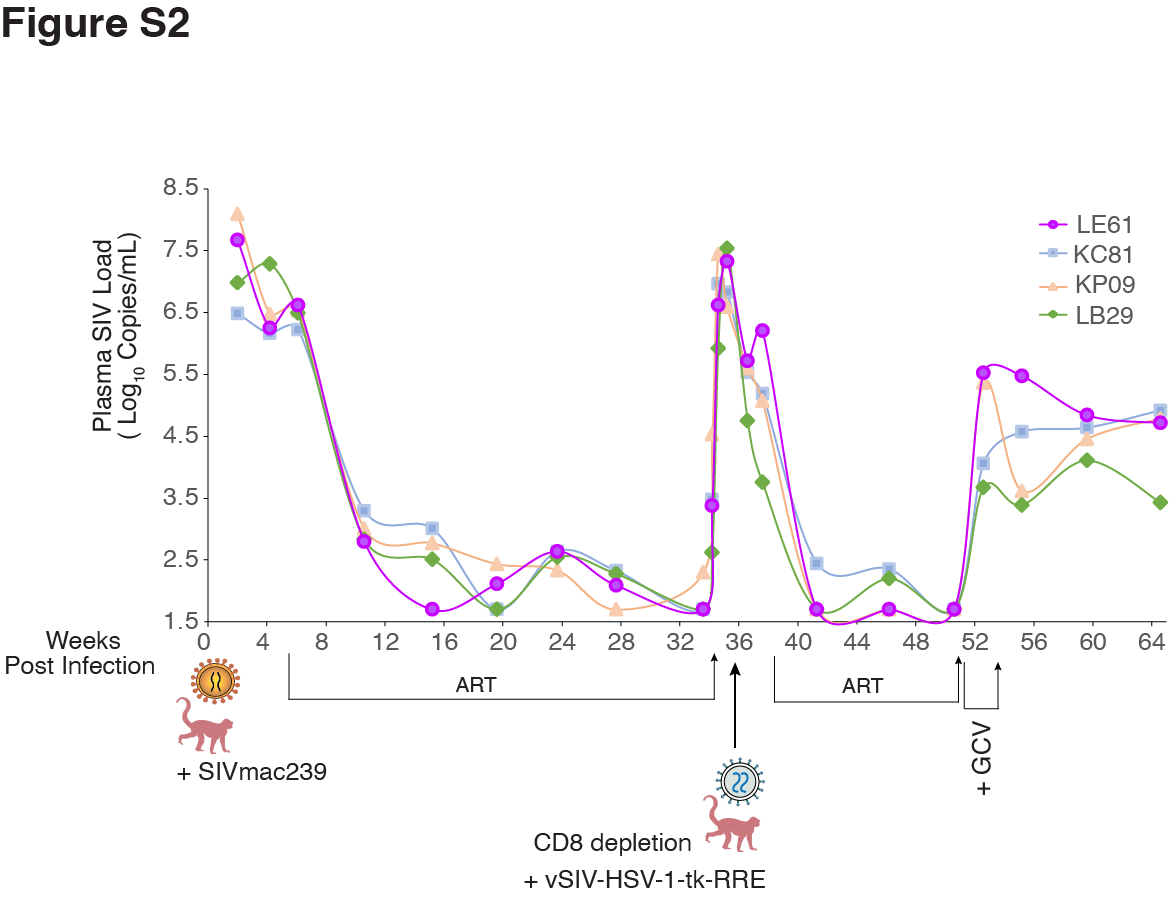


**Figure S2. *In vivo* testing of the SIV Rev-dependent vectors in SIVmac239-infected Indian rhesus macaques.** Shown are plasma viral loads (copies/ml) following SIVmac239 infection, ART, and vSIV-HSV-1-tk-RRE/GCV injection in animals. The infection and treatment regimen followed the similar early course of the Group D animals.


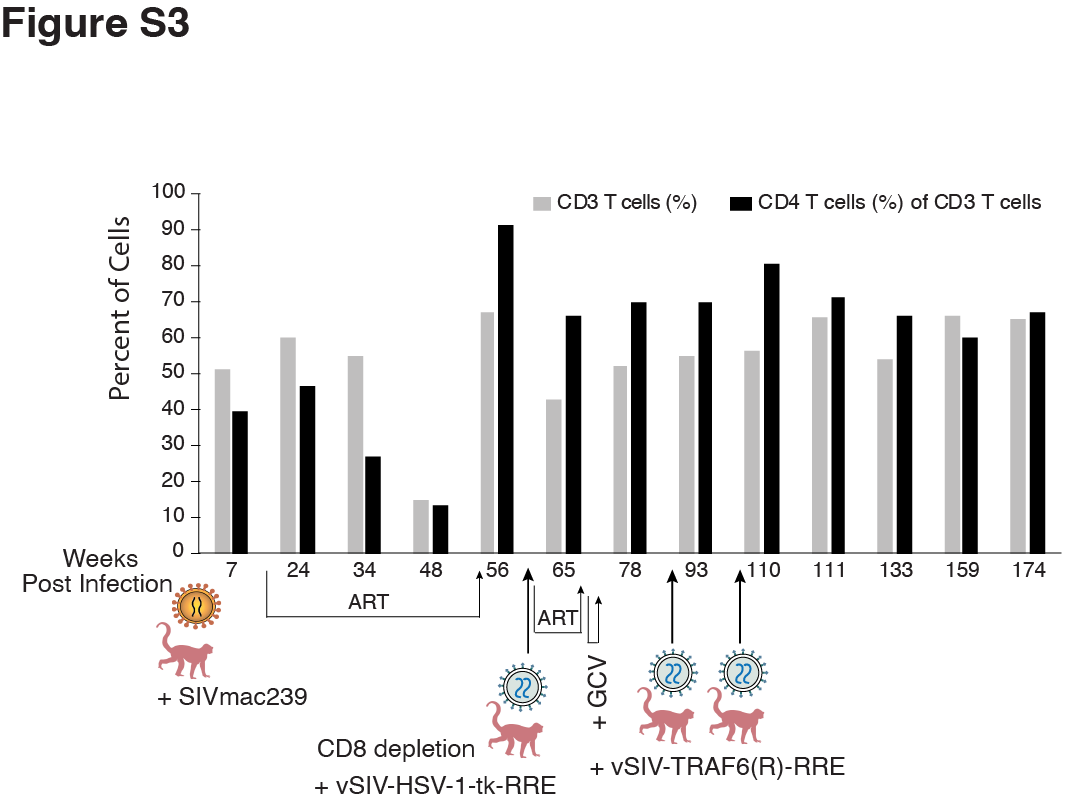


**Figure S3. Quantification of peripheral blood CD3 and CD4 T cells in KC50.** The percentages of CD3 T cells and the CD4 T cells of total CD3 T cells were enumerated and plotted following the time course of SIV infection and treatments of KC50.
